# Evaluation of sonographic detectability of different markers within an in vitro simulation model of the axilla

**DOI:** 10.1007/s00404-021-06085-9

**Published:** 2021-06-17

**Authors:** Selin Guergan, Uta Hoopmann, Carmen Roehm, Bettina Boeer, Regina Fugunt, Gisela Helms, Anna Seller, Mario Marx, Ernst Oberlechner, Andreas Hartkopf, Heike Preibsch, Sara Brucker, Diethelm Wallwiener, Markus Hahn, Ines Verena Gruber

**Affiliations:** 1 Department for Women’s Health, University of Tübingen, Calwerstraße 7, 72076 Tübingen, Germany; 2 Diagnostic and Interventional Radiology, University of Tübingen, Tübingen, Germany; 3 Department of Plastic, Reconstructive and Breast Surgery, Elblandklinikum Radebeul, Radebeul, Germany

**Keywords:** Breast cancer, Targeted axillary dissection, Clip-marking, Sonographic detectability, Lymph node

## Abstract

**Purpose:**

Clip-marking of axillary lymph nodes with initial biopsy-confirmed metastasis is required for targeted axillary dissection (TAD), which includes sentinel lymph node dissection (SLND) and selective localization and removal of the clipped targeted lymph node. There have been several studies which examined the feasibility of TAD in routine clinical use. In this context, the optimal clip visualisation was noted as one of the crucial limiting factors. We, therefore, evaluated the sonographic detectability of 10 different commercially available markers within an in vitro model simulating the anatomical composition of the axilla.

**Methods:**

In this standardised model consisting of porcine fat with 30 mm thickness, the visibility of a total of ten markers was analysed in all 3 planes (parallel, diagonal, orthograde) with wire guidance and then classified into either “visibility good”, “visibility moderate” or “visibility poor” with regard to the alignment of the transducer. Additionally, “real-life conditions” were simulated, in which the markers were searched without any wires guidance.

**Results:**

It was observed that, while not all markers are detectable in fatty tissue, markers with spherical shape (non-embedded Inconel or Nitinol) or rectangular-shaped Titanium markers with embedded material have a clear advantage. 3D-shaped markers can always be detected in all three axes, which is of particular importance in the axilla with its pyramid shape and fatty tissue.

**Conclusion:**

The shape and the embedding of the material play a crucial role for visibility and efficacy of the marker, as reliable marking of suspicious and pathological axillary lymph nodes is essential for TAD.

## Introduction

The involvement of the lymphatic system and its management has played an important part in the treatment of breast cancer over the last 100 years. From radical axillary dissection as favoured by Halsted up to sentinel lymph node biopsy (SLNB), we have seen a significant surgical de-escalation over the past decades [1, 2]. This is because radical axillary dissection is associated with a higher risk of complications such as lymphoedema, pain, paraesthesia, and shoulder dysfunction and, therefore, lacks benefit, when compared to SLNB [2].

Recently, several studies evaluated the different surgical approaches with regard to morbidity as well as false-negative rates (FNRs) of SLNB and axillary lymph node dissection (ALND). It should be noted that cN + patients, namely patients with lymph nodes suspicious of metastases confirmed by biopsy, who received primary systemic therapy, showed a complete remission of the metastatic disease (ypN0) in 40–74% of the cases [2–4].

The ACOSOG Z1071 and SENTINA trials presented FNRs higher than 10% in these ypN0 patients, who received SLNB [3, 5]. However, when the initially suspicious lymph node was clipped, the FNR was reduced to 6, 8%. Caudle et al. demonstrated that a targeted axillary dissection (TAD), which consists of SLNB and marking of the targeted lymph node with iodine 125 seeds, reduces the FNR to 2% [6]. The MARI study also used radioactive iodine seeds to mark the axillary lymph nodes [7]. Since utilisation of radioactive iodine seeds is not authorised in Germany, Hartmann et al. conducted a feasibility study for wire localisation of clip-marked axillary lymph nodes, in which they concluded that this method was not appropriate for routine clinical use and optimal clip visualisation was one of the crucial limiting factors [2]. Superparamagnetic iron oxide (SPIO) or sterile carbon suspension (Spot) are further tracers for lymph node detection. In case of tattooing suspicious lymph nodes with sterile carbon suspension before neoadjuvant chemotherapy intraoperative visual inspection of the lymph nodes is imperative [8]. As a result of these limitations, a consensus regarding TAD and the number of lymph nodes that should be marked before neoadjuvant chemotherapy is not yet reached. Consequently, German Gynaecological Oncology Group recommends TAD at the moment only in medical studies and not as a standard of care [9]. Although there have been several studies comparing biopsy marker visibility in breast tissue, due to the novelty of clip-marking the axillary lymph nodes, further studies are required to determine the optimum marker for axilla. Therefore, we evaluated the sonographic detectability of different markers within a simulation model of the axilla.

## Materials and methods

We developed an in vitro simulation model of the axilla to analyse the sonographic detectability of different markers in the axilla.

Therefore, we built up a model consisting of porcine fat with 30 mm thickness, to simulate the anatomical composition of axillary tissue. It was approximately 40 cm long and 20 cm wide. In this setting depth, consistency as well as the composition of axillary fatty tissue was taken into account.

Ten different commercially available markers that are in general clinical use were placed into the model with a standardised distance to the surface of 20 mm at room temperature. The markers were then searched in all three dimensions (parallel, diagonal and orthograde to the transducer) using a 12 MHz ultrasound transducer (Phillips EPIG 7Q) by three independent examiners (SG, UH, MH). All examiners were physicians specialised in breast diagnostics with the different qualification of DEGUM levels by the German Society of Ultrasound in Medicine (MH with DEGUM Level III, UH with DEGUM Level II and SG with DEGUM Level I).

In the first part of the study, a wire was placed next to the markers to eliminate possible false-positive results and ensure correct localization.

**Fig. 1:**
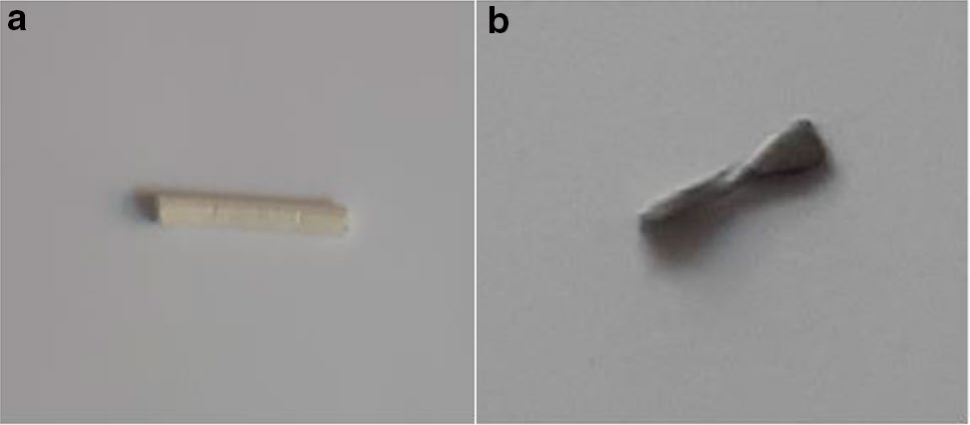
CorMARK® with (**a**) and without (**b**) its embedding material*

**Fig. 2:**
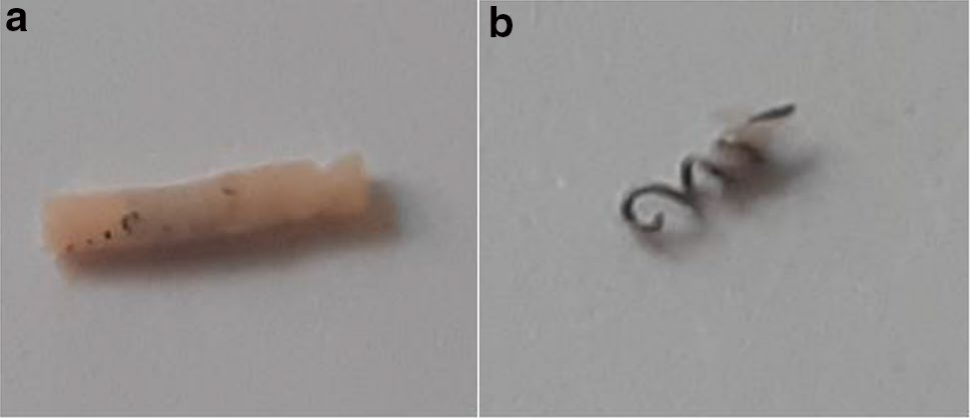
HydroMARK® Shape 3 with (**a**) and without (**b**) its embedding material*

**Fig. 3:**
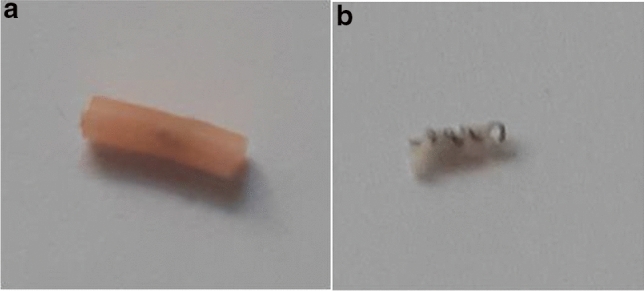
HydroMARK® Shape 4 with (**a**) and without (**b**) its embedding material*

**Fig. 4:**
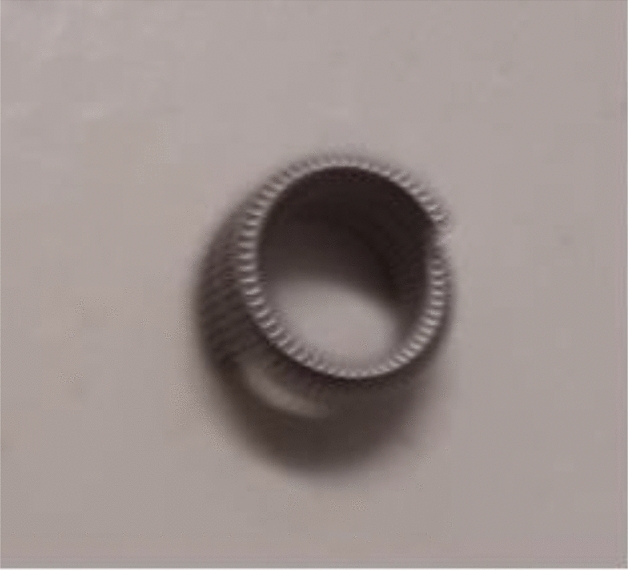
MReye® Coil Marker*

**Fig. 5:**
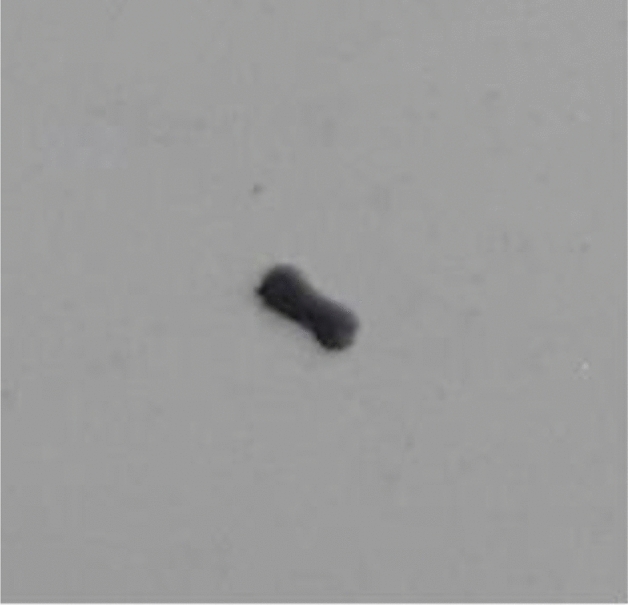
BiomarC®*

**Fig. 6:**
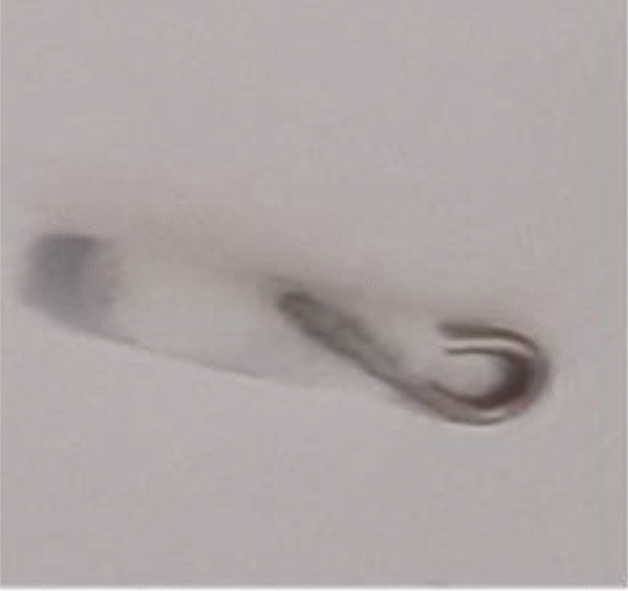
UltraClip® DualTrigger*

**Fig. 7:**
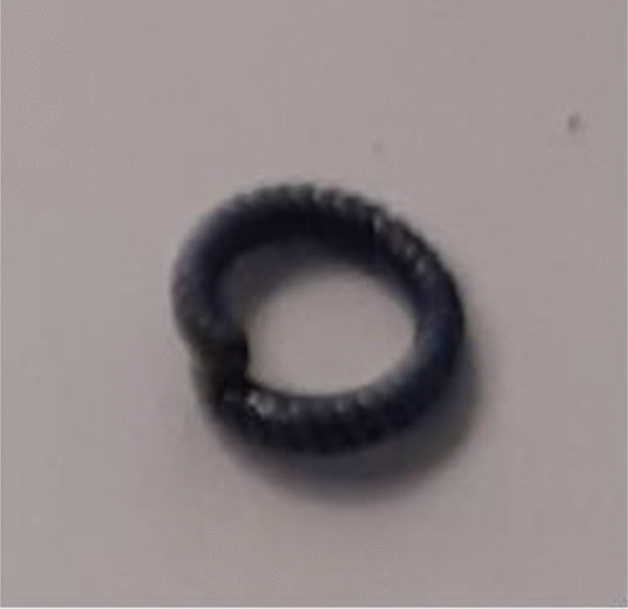
O-Twist-Marker ® *

**Fig. 8:**
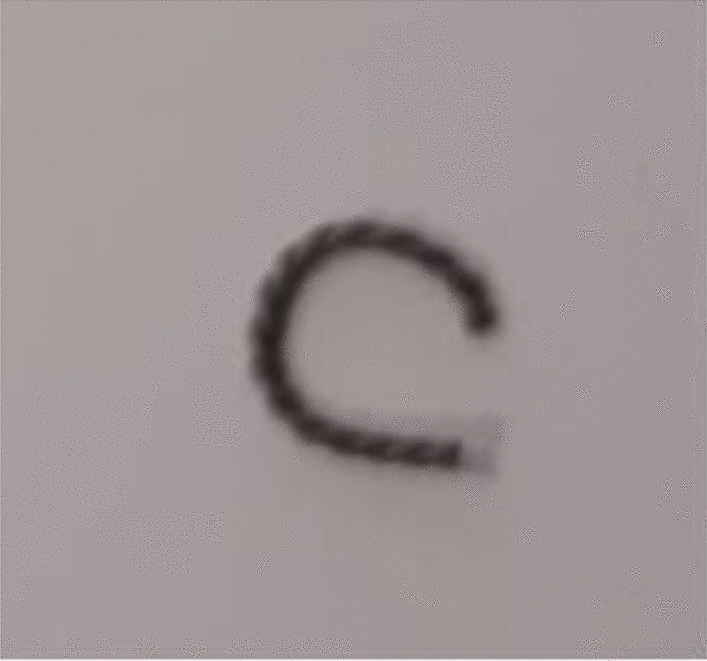
Tumark® Professionel

**Fig. 9:**
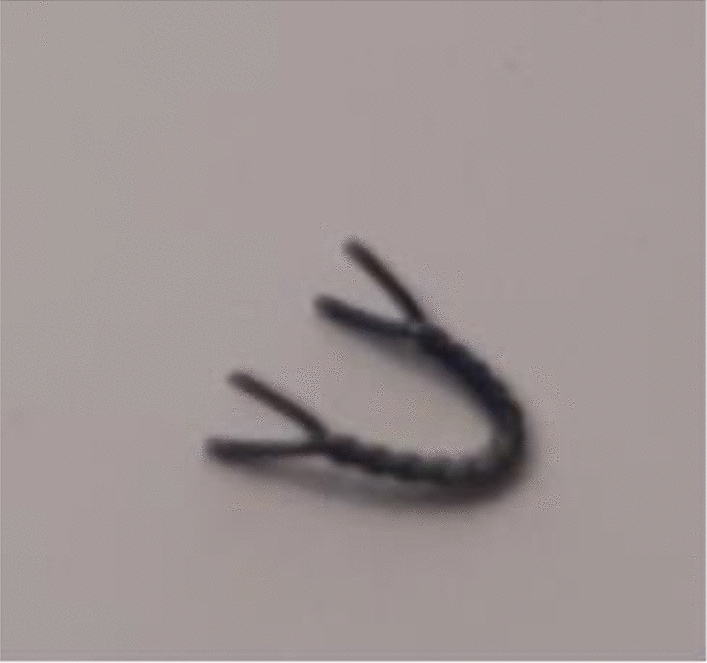
Tumark® Professional* Q-Shape*

**Fig. 10:**
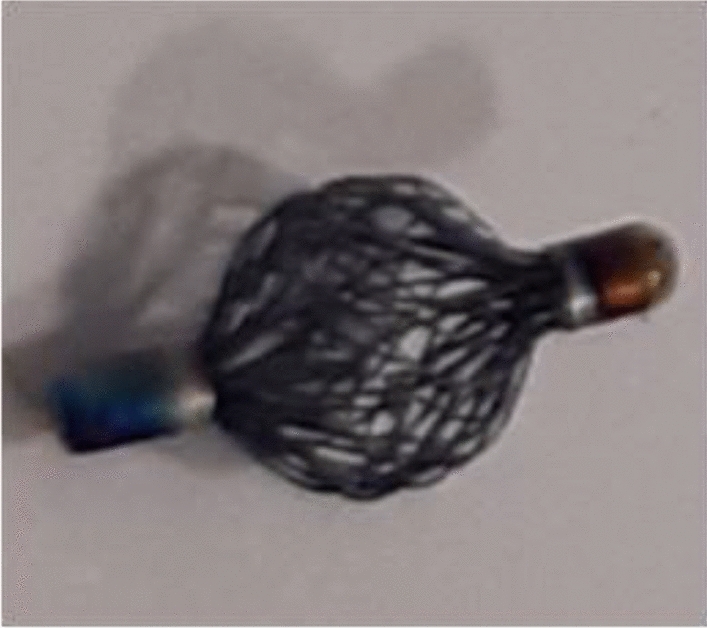
Tumark® Vision*. *All photographs taken by SG

The markers included CorMARK®, HydroMARK® Shapes 3 and 4 and BiomarC® (Mammotome, Leica Biosystems, Wetzlar, Germany), MReye® Coil Marker (Cook Medical, Bloomington, Indiana, USA), UltraClip® Dual Trigger (BARD Biopsy, Arizona, USA), o-Twist-Marker® (BIP Medical, Germany) and several Tumark® clips (SOMATEX Medical Technologies, Germany) (Table 1; Figs. 1, 2, 3, 4, 5, 6, 7, 8, 9, 10). CorMARK® and HydroMARK® clips were also examined after removal of collagen and hydrogel embedding material (Figs. 1b, 2b, 3b) to evaluate detectability of the radiopaque marker material. The visibility of the markers was examined in all 3 planes with and without wire guidance and then classified into either “visibility good”, “visibility moderate” or “visibility poor” with regard to the alignment of the transducer (Table 2). Markers that were detectable in all three planes without any wire guidance were classified as “easy to detect” (visibility good), markers that were only detectable in one or two planes without any wire guidance were classified as “difficult to detect” (visibility moderate). If a marker could not be seen and its placement was only detectable as a result of wire guidance, it was classified as “not detectable” (visibility poor), as wire guidance should be avoided in real-life applications .After all markers were examined with wire in all three planes, the wire was then discarded.

**Table 1:** Overview of examined breast tissue biopsy markers

Clip type	Biopsy Marker	Manufacturer	Radiopaque marker material	Embedding Bioresorbable material	Marker shape	Price per marker (approximately)
Type-1	CorMARK ®	Mammotome, Leica Biosystems	Titanium	Collagen	Rectangular	$ 37
Type-2	MReye ® Coil Marker	Cook Medical	Inconel	None	Round/spherical	$ 108
Type-3	HydroMARK ® Titan Shape 3 (open coil)	Mammotome, Leica Biosystems	Titanium	Polyethylene glycol-based hydrogel (PEG)	Rectangular	$ 30
Type-4	HydroMARK ® Titan Shape 4 (butterfly)	Mammotome, Leica Biosystems	Titanium	Polyethylene glycol-based hydrogel (PEG)	Rectangular	$ 30
Type-5	BiomarC ®	Mammotome, Leica Biosystems	Carbon coated ceramic	None	Rectangular	$ 25
Type-6	UltraClip ® DualTrigger	BARD Biopsy	Titanium	None	Twisting	$ 39
Type-7	O-Twist-Marker ®	BIP	Nitinol (Nickel titanium alloy)	None	Round/spherical	$ 120
Type-8	Tumark ® Professional Q-Shape	SOMATEX Medical Technologies	Nitinol (Nickel titanium alloy)	None	Twisting	$ 85
Type-9	Tumark ® Professional Q-shape	SOMATEX Medical Technologies	Nitinol (Nickel titanium alloy)	None	Twisting	$ 85
Type-10	Tumark ®Vision	SOMATEX Medical Technologies	Nitinol (Nickel titanium alloy)	None	Round/spherical	$ 120

**Table 2:** Comparison of detectability of examined markers

Marker type		Embedded	Alignment of the marker to the transducer			Search test
Clip type	Marker	Embedding material	Parallel	Diagonal	Orthograde	Visibility
Type-1	CorMARK ®	Collagen	Visibility good	Visibility good	Visibility good	Good (detectable)
Type-1a	CorMARK ®	None	Visibility moderate	Visibility poor	Visibility poor	Poor (not detectable)
Type-2	MReye ® Coil Marker	None	Visibility good	Visibility good	Visibility good	Good (detectable)
Type-3	HydroMARK ® Shape 3	Polyethylene glycol-based hydrogel (PEG)	Visibility good	Visibility good	Visibility good	Good (detectable)
Type-3a	HydroMARK ® Shape 3	None	Visibility moderate	Visibility moderate	Visibility poor	Poor (not detectable)
Type-4	HydroMARK ® Shape 4	Polyethylene glycol-based hydrogel (PEG)	Visibility good	Visibility good	Visibility good	Good (detectable)
Type-4a	HydroMARK ® Shape 4	None	Visibility moderate	Visibility poor	Visibility poor	Poor (not detectable)
Type-5	BiomarC ®	None	Visibility moderate	Visibility poor	Visibility poor	Poor (not detectable)
Type-6	UltraClip ® DualTrigger	None	Visibility moderate	Visibility moderate	Visibility poor	Moderate (detectable)
Type-7	O-Twist-Marker ®	None	Visibility good	Visibility good	Visibility good	Good (detectable)
Type-8	Tumark ® Professional Q-Shape	None	Visibility good	Visibility good	Visibility moderate	Moderate (detectable)
Type-9	Tumark ® Professional	None	Visibility good	Visibility good	Visibility moderate	Moderate (detectable)
Type-10	Tumark ®Vision	None	Visibility good	Visibility good	Visibility good	Good (detectable)

Since the examiner is not aware of the placement of the marker in real-time clinical conditions, in the second part of the study, the markers were then discarded into the same standardised simulation model in approximately 20 mm distance to the surface by an assistant and afterwards searched by an examiner without wire guidance to imitate the clinical situation. The detectability of the markers in regard to the alignment of the ultrasound transducer was then classified into either “easy to detect”, “difficult to detect” or “not detectable” (Table 2) within the classification mentioned above. This procedure was repeated by the different examiners (SG, UH, MH). The results of the evaluation were anonymous.

A total of 10 markers were evaluated. The visibility of markers with embedding material was also separately examined after the collagen material was stripped. The shape of the marker, presence of embedding material, alignment of the marker, transducer as well as the radiopaque material of the marker were crucial for the detectability of the markers.

## Results

It was observed that markers with round or spherical shapes such as Tumark® Vision, O-Twist Marker®, and MReye® Coil Marker were easily detectable regardless of the alignment of the marker and the transducer. This trend was also reflected in the simulation of the “real-life” conditions regarding the detectability of the markers, in which the markers were searched for and found easily in all three axes (Table 2).

CorMARK® and HydroMARK® clips with embedding material were in all cases—independent of the positioned axes—easily detected. Furthermore, they proved to be easily visible during the search test. However, it was observed that, after these clips were stripped of their embedding material, detectability was very difficult or not possible.

It was also observed that rectangular markers without any embedding material, such as BiomarC® and, CorMARK® and HydroMARK® markers without their embedding material, were nearly impossible to detect in any of the axes and could not be found during the search test. Markers with twisting shapes such as UltraClip® DualTrigger, Tumark® Professional Q-Shape and Tumark® Professional also proved difficult to detect in the fatty tissue. Although Tumark® Professional Q-Shape and Tumark® Professional were easily visible in parallel and diagonal axes, in the third dimension, the orthograde axis, they were barely seen (Table 2).

It was observed that marker and transducer alignment affected marker visibility. Differentiation the marker from the surrounding tissue was especially diminished in the orthograde axis. This effect was particularly notable in markers with rectangular shapes.

The radiopaque marker material was influential in distinguishing the marker from the surrounding tissue. Markers made of nitinol, a nickel titanium alloy, were more easily detected. Titanium markers were only visible with the help of the embedding material. Inconel® coil, which is a superalloy of nickel, chromium and iron, could be easily found using ultrasound.

## Discussion

After it was proven in the ACOSOG Z1071 trial that placement of a clip marker in the lymph node during initial biopsy and localization of the marker during the SNB caused the FNR rate to drop from 12, 6% to 6, 8%, the concept of targeted axillary dissection (which combines SNB with removal of the clipped node) gained importance [3]. Caudle et al. observed an even lower FNR rate of 2% [6]. However, while many studies [5–8, 10] agree with the possible benefits of a TAD approach for the patient, such as less invasive surgery and a decrease in possible complications as a result, namely lymphoedema, pain, paraesthesia and shoulder dysfunction, the question of which type of marker should be used in the axilla remained open. In many trials [2, 6, 10, 11], which have investigated the efficacy and feasibility of the clipped lymph nodes, visibility and preoperative localization of the used markers played a crucial role. Therefore, missed nodes and markers may cause undertreatment and, thus, may affect regional recurrence.

Since the implementation of markers for breast lesions was introduced in the 1990s, many different markers have been developed and evaluated in breast tissue [11–13]. However, the sonoanatomy of the breast and axilla is drastically different, as the axilla consists mainly of fatty tissue. Therefore, we used a model equivalent to the axillary tissue to investigate if alignment of the different markers to the transducer influenced marker visibility. Congruent to the dominantly fatty tissue of the axilla our model was composed of porcine fat. Our results indicate that positioning of the markers diagonal or parallel to the transducer, did not affect their visibility. However, the detectability of some markers was affected in the orthograde axis. Rueland et al. found that 3D markers such as Tumark® Vision were easily detectable in both axes [13]. Though, this study consisted of investigation of a sole marker and a third axis was not included. There are no further studies up to now which compare marker positioning.

**Fig. 11:**
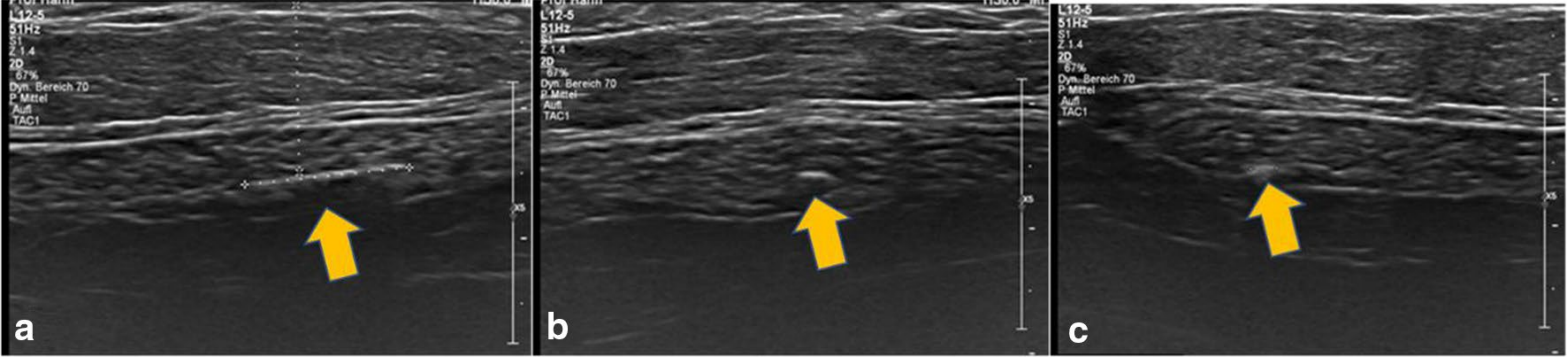
CorMARK ® with embedding material seen in parallel (**a**), diagonal (**b**) and orthograde axes (**c**)

**Fig. 12:**
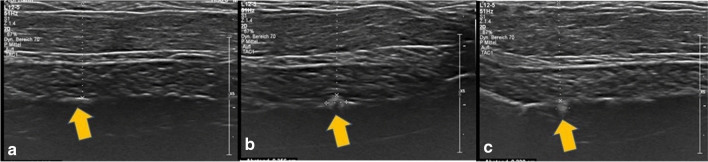
HydroMARK® Shape 4 with embedding material seen in parallel (**a**), diagonal (**b**) and orthograde axes (**c**)

**Fig. 13:**
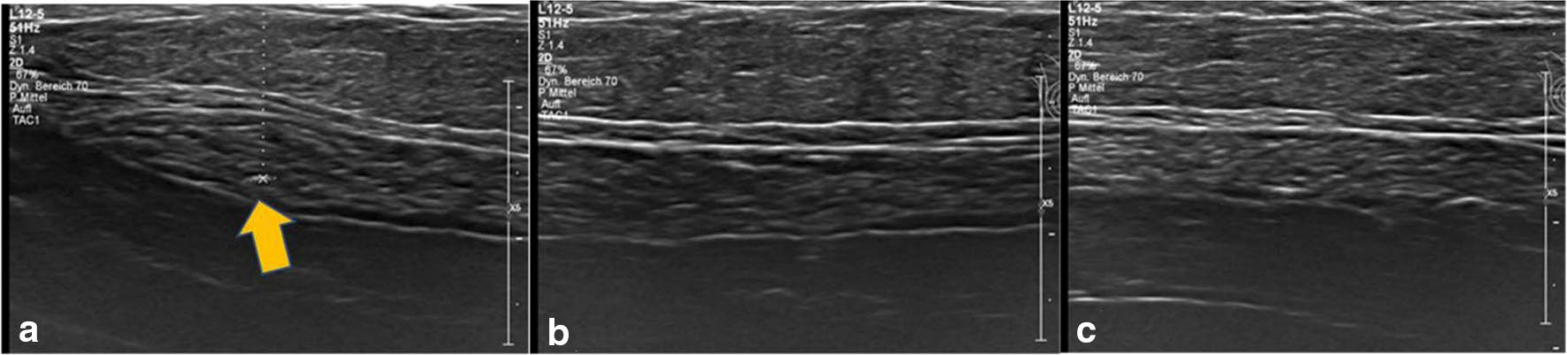
CorMARK® without the embedding material seen in parallel axis (**a**), but not seen in diagonal (**b**) and orthograde axes (**c**)

**Fig. 14:**
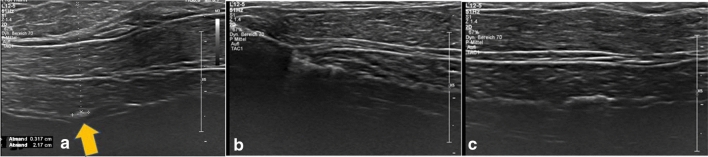
HydroMARK® Shape 4 without the embedding material seen in parallel (**a**), but not seen in diagonal (**b**) and orthograde axes (**c**)

In our study, the orthograde axis proved a particular challenge. As a result of its pyramid shape, the axilla requires markers which are not only visible in two axes, but in all three. Furthermore, markers with rectangular shapes without embedding material were not distinguishable from the surrounding tissue (Table 2). While it was observed that the spherical-round markers were particularly visible, as discussed later, the rectangular makers showed a particular increase in visibility when surrounded by an embedding material. When this material was removed, as seen in examples of HydroMARK ® Shapes 3 and 4 and CorMARK®, the visibility of the marker was diminished (Figs. 1, 3, 11, 12, 13, 14).

The detectability of the marker with ultrasound is especially important to evaluate the clinical response during neoadjuvant chemotherapy. Pinkney et al. noted that the sonographic visibility of the different breast makers (Gelmark UltraCor™, HydroMARK®, SenoMark™ UltraCor™, UltraClip® Enchanced Coil, SecurMark®) changes over time. While the visibility of other markers diminished significantly over time, HydroMARK® remained visible during all 12 weeks of the trial. This was due to physical and chemical properties of polyethylene glycol hydrogel, which attracted water molecules and increased visibility [14]. The efficacy of such embedding materials was observed in our study with CorMARK ® and HydroMARK ® with their embedding material remaining detectable in all three axes. As a result of self-expanding material in the biopsy cavity, hyperechoic material was easily in detected in fatty tissue. However, as Banys-Paluchowski and Gruber et al. noted in their publication, the hydrogel may lead to cavity formation containing mucoid material and pseudocysts and these changes may be reported falsely as a positive lymph node [15].

A further challenge of the detectability of the marker is tissue quality [15, 16]. Fibrosis of the lymph nodes and the surrounding tissue after neoadjuvant chemotherapy can make sentinel node biopsy relatively unreliable [3], and secondary dislocation of the marker may occur. Migration of the clip marker in the biopsy track, floating due to haematoma and change in clip site due to the resorption of air in the biopsy cavity are also sources of error which may result in secondary dislocation. Preoperative ultrasound localization of the marker after neoadjuvant chemotherapy may fail in up to 28% of cases [11, 15]. If the marker is undetectable with ultrasound before planned TAD, mammography of the axilla is not a feasible option, since axilla cannot be accurately scanned with a mammography. MRI or CT scan are then required to locate the marker, which results in additional costs and additional radioactive exposure in the case of CT scan. The patient may require a second invasive procedure, reducing patient comfort and work flow efficacy as well as increasing costs [12].

**Fig. 15:**
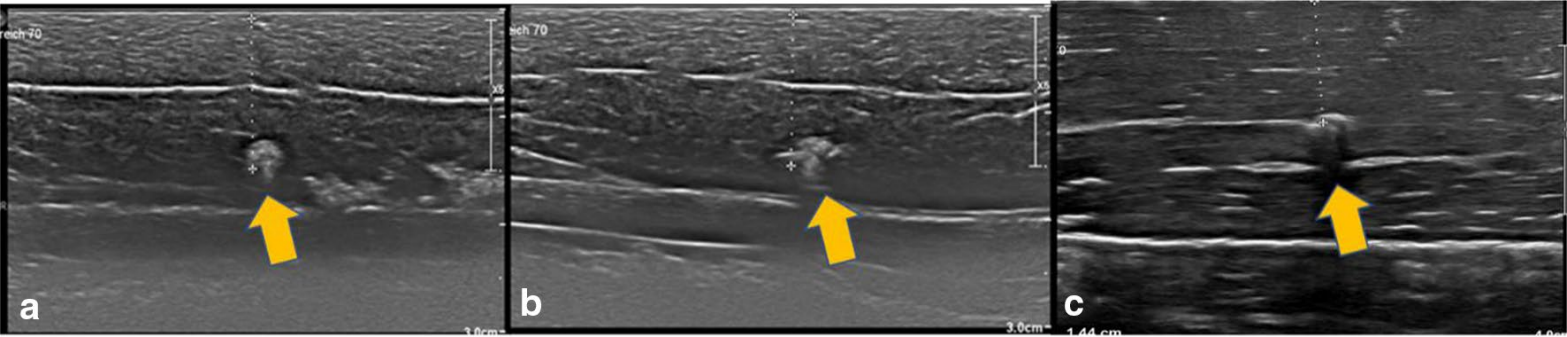
Tumark® Vision seen in parallel (**a**), diagonal (**b**) and orthograde axes (**c**)

**Fig. 16:**
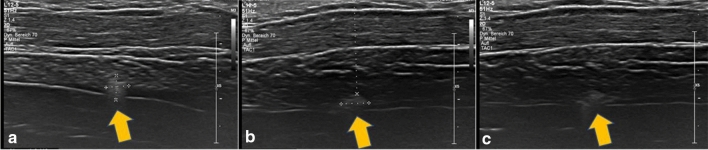
MReye® Coil Marker seen in parallel (**a**), diagonal (**b**) and orthograde axes (**c**)

**Fig. 17:**
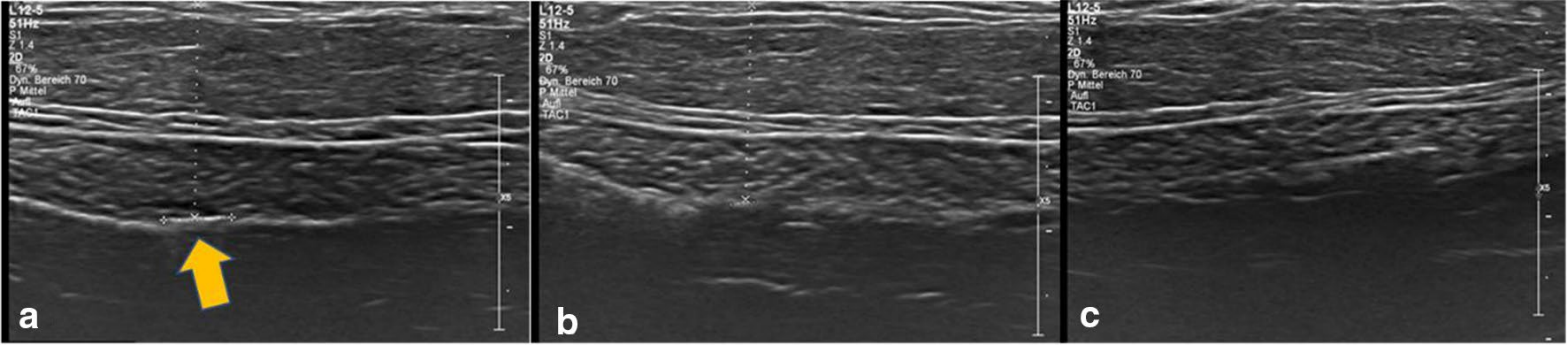
BiomarC® seen in parallel (**a**), but not seen in diagonal (**b**) and orthograde axes (**c**)

**Fig. 18:**
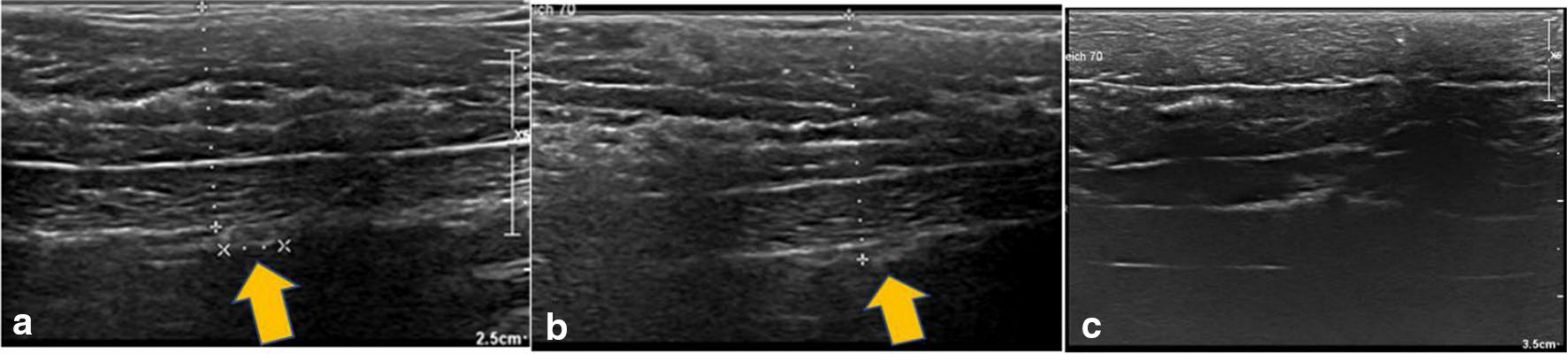
UltraClip® DualTrigger seen in parallel (**a**), diagonal (**b**), but not in orthogonal (**c**)

As noted above, the shape of the marker influences visibility. Spherical markers such as MReye ® Coil Marker (Cook Medical), o-Twist-Marker ® (BIP) and especially Tumark ® Vision (SOMATEK) (Figs. 4, 7, 10) retained their visibility in our standardised simulator. While lacking embedding material, these markers showed a distinct advantage in comparison to UltraClip ® or BiomarC®, which are rectangular markers without any embedding material in fatty tissue (Figs. 15, 16, 17, 18). Detecting markers with twisting shapes such as UltraClip ® DualTrigger, Tumark ® Professional Q-Shape, Tumark ® Professional and HydroMARK ® without their embedding material is difficult (Table 2). It can be hypothesised that the greater volume and the three-dimensional shape of the marker contribute to the visibility. Rueland et al. observed in their study that 3D shapes showed stability in breast tissue [13]. The self-expansion of these markers contributes to this phenomenon, which is underlined with lower displacement rates [14]. While Nguyen et al. have argued that spherical shapes such as Tumark® Vision without embedding gel retain their visibility following neoadjuvant chemotherapy, Pinkney and Shah showed that HydroMARK ® clips with embedding material retain and improve their visibility during 12 weeks of follow-up [11, 14]. To determine the effect of the embedding material on the visibility of the coil, we stripped the gel from coil manually and examined the visibility of the marker. We determined that markers without a spherical shape and without embedding material are clearly disadvantageous.

Another factor in the detectability of the marker was the radiopaque marker material. Markers which contained nickel–titanium alloys were much easier to detect than mere titanium markers. However, this may result in a rare complication with an allergic reaction to the nickel component [17]. Patients with nickel allergy should be informed beforehand of possible complications. It should also be noted that, as can be seen in the examples from the Tumark family (Figs. 8, 9, 10), which were all made up of nitinol, although the radiopaque material shows a fairly good visibility, only Tumark Vision ® with its spherical shape was seen in all three axes (Table 2; Fig. 15). Ergo, it can be surmised that, when it comes to the factors determining the visibility, the shape of the marker plays a more decisive role than the material.

**Fig. 19:**
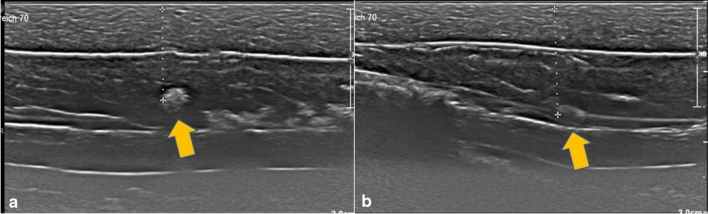
Comparison of Tumark® Vision (**a**) and Tumark® Professional (**b**) in parallel axis

Although we examined different markers to determine the best possible options for TAD, it should be noted that our study was performed in a simulation model and not in vivo. The possible anatomical differences amongst the axilla of the patients, such as muscles, nerves, thoracodorsal bundle and the lymph nodes varying in structure and depth, could not be taken into account in our study. To make the ultrasound images comparable, all markers were placed in approximately the same depth. Utilisation of one simulation model with possible puncture channels may have influenced the visibility of some markers. Additionally, the wire that was placed to ensure correct localisation of the marker is not practicable in real-life conditions. Therefore, only clearly visible markers are suitable for TAD Fig. 19.

Further studies that focus on the issue of possible migration of the markers during neoadjuvant chemotherapy are of interest. Although primarily placed correctly in the targeted lymph node (TLN), downstaging of the metastatic lymph node with its morphological changes such as nodal shrinkage and fibrosis may lead to clip dislocation dependent on the used clip material [3].

Results in prospective and multicentric studies, such as the ongoing AXSANA trial of EUBREAST group, which examines the feasibility and performance as well as the mortality and morbidity of TAD, will contribute to the path of de-escalation in axilla surgery [18].

## Conclusion

While not all markers are detectable in fatty tissue, markers with spherical shape and rectangular-shaped markers with embedded material have a clear advantage. The material of the radiopaque material plays a lesser role in visibility. 3D-shaped markers can always be detected in all three axes, which is of particular importance in the axilla with its pyramid shape and fatty tissue.

Since the significance of TAD is increasing due to low FNR and less possible complications and, thus, increased patient comfort, physicians should be informed about the detectability of the marker they use.
